# Health literacy, eHealth literacy and their association with burden, distress, and self-efficacy among cancer caregivers

**DOI:** 10.3389/fpsyg.2024.1283227

**Published:** 2024-02-16

**Authors:** Chloe Moore, Pamela Gallagher, Simon Dunne

**Affiliations:** School of Psychology, Dublin City University, Dublin, Ireland

**Keywords:** health literacy, cancer caregiver, cancer, oncology, caregiver

## Abstract

**Purpose:**

Health literacy skills are vital for cancer caregivers in helping cancer survivors to navigate their diagnosis, treatment, and recovery but little is known. This study explored health literacy and eHealth literacy among cancer caregivers and the relationship between health literacy/eHealth literacy and potential associated factors.

**Methods:**

Informal caregivers who had cared for an individual with cancer completed a survey which collected demographic data and measured caregiver health literacy, eHealth literacy, self-efficacy, burden, and distress.

**Results:**

Seven percent of caregivers had inadequate health literacy. Caregivers scored lowest on health literacy domains related to caregiver social support, information seeking and understanding care recipient preferences. eHealth literacy was associated with self-efficacy and burden while, different health literacy domains were associated with burden (‘*Understanding care recipient needs and preferences*’), self-efficacy (‘*Cancer-related communication with the care recipient*’ and ‘*Understanding care recipients needs and preferences*’) and distress (‘*Proactivity and determination to seek information’*, ‘*Understanding care recipient needs and preferences’*, ‘*Understanding the healthcare system*’).

**Conclusion:**

Findings highlight key areas of need regarding cancer caregiver health literacy which future research can target. Given the observed relationship between aspects of health literacy and burden, distress and self-efficacy future work could be carried out on how to alleviate high levels of burden and distress and how to enhance self-efficacy among cancer caregivers by addressing health literacy skills.

**Implications for cancer survivors:**

Findings from this study will inform the development of health literacy interventions to support caregivers to build their health literacy skills and enable this group to better support cancer survivors as a result.

## 1 Background

Caregivers though often overlooked, play a pivotal role in the diagnosis, treatment and recovery of cancer survivors (Bevan and Pecchioni, 2008). Cancer caregivers can be defined as family members or close friends who provide informal care to a loved one with cancer: this informal care is typically unpaid and primarily takes place at home (Kent et al., 2016). The roles and responsibilities that caregivers are required to carry out when caring for an individual with cancer is dependent upon the diagnosis and severity of the illness (LeSeure and Chongkham-ang, 2015). However, caregivers are often actively involved in communicating and sharing health information with healthcare professionals, the care recipient and within wider support networks (i.e., other family members) (Bevan and Pecchioni, 2008). Caregivers also play a role in making health-related decisions, finding relevant health information and interpreting such information (Hubbard et al., 2010; Laidsaar-Powell et al., 2018). As such, cancer caregiver health literacy may be of particular importance in this context.

Health literacy can be defined as the personal characteristics and social resources needed to access, understand and use information and services to make health related decisions (Sørensen et al., 2012). It is a complex construct consisting of individual characteristics and broader healthcare community factors (Yuen et al., 2016). Yuen and colleagues (Yuen et al., 2016) suggest that cancer caregiver health literacy is comprised of six key elements; access to information, understanding of information, relationship with the care recipient, relationship with the healthcare providers, managing challenges of caregiving and support systems, all of which cancer caregivers would be faced with day-to-day when navigating the caregiving role. Furthermore, caregiver eHealth literacy may also be of particular importance when caring for an individual with cancer. eHealth literacy can be defined as an individual’s ability to obtain, understand, exchange and evaluate health information from the internet (Vasquez et al., 2022). Caregivers often use online platforms to obtain health information regarding the care recipient’s cancer and treatment and medical options and to connect with others who have had similar experiences (Chen, 2014). However, caregivers’ ability to engage in these online behaviors is dependent upon their eHealth literacy skills (Vasquez et al., 2022).

There are multiple studies which have captured challenges that caregivers face when caring for a loved one with cancer (Petronio et al., 2004; Bayen et al., 2017; Goldsmith and Terui, 2018) which are relevant to health literacy. Caregivers have reported difficulties in comprehending information given at medical appointments and in obtaining information from health professionals regarding the prognosis, treatment and recovery of the care recipient (Petronio et al., 2004). A recent review indicated that caregiver involvement is vital in helping cancer patients with health-related decision making, that cancer patients prefer caregivers to be active collaborators regarding their health decisions throughout the cancer trajectory and that cancer patients like to discuss treatment options with caregivers (Cincidda et al., 2023). Complex tasks such as medication management have also been identified as a difficulty for caregivers (Goldsmith and Terui, 2018). Other responsibilities associated with caring that caregivers have indicated are challenging, include symptom management and providing emotional support (DuBenske et al., 2010; Yuen et al., 2016; Bayen et al., 2017).

The way in which caregiver health literacy has been measured and reported varies widely (Moore et al., 2021). Across general informal caregiver populations, inadequate health literacy levels vary from 0 to 42.9% (Yuen et al., 2018). Similarly, a recent study which explored inadequate/adequate health literacy among cancer caregivers found that the majority of their sample had adequate health literacy (Oakley-Girvan et al., 2023). However, in contrast, a different study using a similar measurement tool found that the majority of cancer caregivers had inadequate health literacy (Gabriel et al., 2021). In addition to dichotomous adequate/inadequate measures of health literacy, health literacy has also been measured using domain-based comprehensive measurement tools such as the Health Literacy Questionnaire (HLQ) (Osborne et al., 2013) and the Health Literacy of Caregivers Scale Cancer (HLCS-C) (Yuen et al., 2018). Caregivers of individuals with a chronic illness scored lowest in domains regarding social support and managing their own health (Cianfrocca et al., 2018) while cancer caregivers using the HLQ scored lowest in appraisal of health information and managing their health (Høeg et al., 2021). Low health literacy can have a negative impact on the quality of care the caregiver is able to provide, yet few studies have focused on cancer caregiver health literacy specifically, with a recent systematic review highlighting the overall dearth of empirical research in the area (Moore et al., 2021). The HLCS-C has been used among cancer caregivers once and found that caregivers scored lowest overall on the self-care domain (Wittenberg et al., 2019).

In addition to the lack of research regarding cancer caregiver health literacy levels, there is also little known about the potential factors associated with cancer caregiver health literacy. Individual characteristics such as caregiver age, gender or education levels may be associated with cancer caregiver health literacy; however, there is currently no consensus in the literature as to what factors are associated with cancer caregiver health literacy. Previous studies have found associations between cancer caregiver health literacy and patient depression and health-related quality of life (Høeg et al., 2021), caregiver communication type (Wittenberg et al., 2019), cancer survivor health literacy (Kayser et al., 2015; Lim et al., 2019) and caregiver coping styles (Gelkopf et al., 2019). One study which looked at eHealth literacy specifically among cancer caregivers, found that caregiver eHealth literacy was associated with education levels, race, household income, access to the internet and caregiver/cancer survivor health status (Song et al., 2017). eHealth literacy was also found to be associated with the size of one’s social network, their involvement in seeking a second opinion and their awareness of different treatment options (Song et al., 2017).

Furthermore, there are other factors which may also be important to consider regarding cancer caregiver health literacy. For example, caregiver self-efficacy may also be essential to consider in the context of caring for an individual with cancer (Hendrix et al., 2016). A previous study carried out with informal caregivers of individuals with dementia found that carers with higher health literacy were more likely to report higher self-efficacy (Efthymiou et al., 2022). Patients with higher health literacy have also been found to have higher self-efficacy in carrying out the tasks necessary to care for themselves (von Wagner et al., 2009). Additionally, a previous study with caregivers of those with Alzheimer’s found that self-efficacy mediates the relationship between caregiver preparedness and burden (Phongtankuel et al., 2023). Indicating that self-efficacy and caregiver preparedness which could also be related to caregiver health literacy skills are modifiable and could aid in alleviating caregiver burden (Phongtankuel et al., 2023). However, currently, little is known about the relationship between health literacy and self-efficacy in the context of cancer caregivers.

The prevalence of caregiver burden and distress amongst cancer caregivers as a result of the caregiving role is well documented across the literature, however, little is known about the relationship between caregiver health literacy and burden and distress, despite the importance of health literacy skills in navigating the caregiver role. Cancer caregivers provide support both emotionally and physically throughout the cancer journey, whilst dealing with their own emotions and needs (Cincidda et al., 2023). At times, cancer caregivers and patients may disagree about issues such as the sharing of information relating to the patient’s cancer and treatment (Cincidda et al., 2023), which can lead to increased burden and distress for caregivers. Caregiver health literacy may impact on burden and distress, which may negatively impact both the caregiver and care recipient. For example, lower cancer caregiver health literacy was found to be associated with higher caregiver burden (Venetis et al., 2014). Caregivers also experience burden that is specific to different aspects of the caregiver role. For example, information-seeking burden has been found to be associated with information overload among caregivers (Kim, 2021), an aspect of caregiving which health literacy skills may help caregivers to address. Additionally, low caregiver health literacy may be associated with distress levels. To the best of the authors knowledge no study has examined the relationship between health literacy and distress among cancer caregivers. However, one study among colorectal cancer survivors found that lower patient health literacy was associated with higher distress (Husson et al., 2015). Inadequate information among caregivers has been found to be associated with higher distress (Fried et al., 2005). Family caregivers have also reported feeling distress as a result of poor communication with family members (Li and Loke, 2014). It may be particularly important to investigate the relationship between health literacy and distress among caregivers given that many of these skills discussed above are related to health literacy.

Additionally, it may be useful to further investigate the relationship between health literacy, self-efficacy, burden, and distress. In addition to being related to health literacy, self-efficacy has also been found to be related to both burden (Gallagher et al., 2011; Phongtankuel et al., 2023) and distress (Chirico et al., 2017). It has also been suggested that many factors may mediate the relationship between health literacy and health outcomes, one of which may be self-efficacy (Squiers et al., 2012). In particular, a framework developed by Paasche-Orlow and Wolf (2007) proposing the causal pathways linking health literacy to different health outcomes suggests that self-efficacy may mediate the relationship between health literacy and health outcomes. While this framework was developed for patients, subsequent research has looked at the mediating role of self-efficacy among cancer caregivers, finding that self-efficacy partially mediated the relationship between communication with healthcare professionals and distress, and fully mediated the relationship between caregiver trust in health professionals and distress levels (Oh, 2017). Communication with healthcare professionals and trust in healthcare professionals are factors similar to subcomponents of caregiver health literacy, for example, the HLCS-C measurement tool includes domains on caregivers ‘Active engagement with healthcare professionals’ and feeling ‘Supported by healthcare professionals’. However, to our knowledge, no study has empirically examined these relationships. Understanding whether variables such as cancer caregiver health literacy, comprised of several skills which are vital to the caregiving role and which have been shown to be associated with burden and distress in the literature, are mediated by caregiver self-efficacy may help to define the mechanisms underlying the relationship and further the understanding of which variables may be useful to target in health literacy interventions for cancer caregivers.

To provide optimal support to cancer caregivers, and in turn to those with cancer, insight into cancer caregivers’ health literacy levels and the potential relationship between health literacy and relevant factors is pressing. As such, this exploratory study has three key objectives-

1. To examine health literacy and eHealth literacy among cancer caregivers to further the understanding on health literacy/eHealth literacy levels and potential areas of need within this group.
2. To investigate factors associated with cancer caregiver health literacy and eHealth literacy.
3. To explore the mediating role of self-efficacy between health literacy/eHealth literacy and the key outcomes of burden and distress for cancer caregivers.

This knowledge may help to identify ways to better support cancer caregivers to ensure that they provide the best quality of care for cancer survivors.

## 2 Methods

### 2.1 Design

The data reported here are from a cross-sectional survey designed with guidance from a project steering group of cancer caregivers who have cared for individuals with different cancer types.

### 2.2 Participants

Informal caregivers, over the age of 18 who were currently providing care or had previously cared for an individual with cancer in the last 5 years were eligible to take part. Caregivers were recruited via multiple sampling approaches with the aim of maximizing the number of caregivers we could reach. This included a recruitment call-out across social media platforms Twitter and Facebook, targeted requests to relevant organizations to share recruitment advertisements across social media and within their organizations, and a press release targeting national and local news media outlets.

### 2.3 Measures

The survey consisted of a brief socio-demographic section including questions on; Gender, age, education level, employment status, relationship to the care recipient, cancer type, cancer stage, treatment type, time spent caring, and health status of caregiver. The survey also included the following six measures:

The Health Literacy of Caregivers Scale-Cancer (HLCS-C) (Yuen et al., 2014) is a 46 item measure developed specifically to measure cancer caregiver health literacy across 10 domains. Higher scores on each domain indicated higher health literacy and fewer health literacy needs in that specific domain. In this study, the Cronbach’s alpha ranged from 0.83–0.94, similar to a previous study which reported good internal consistency across the domains (0.78–0.92) (Yuen et al., 2018).

The 6-item Cancer Health Literacy Test (CHLT-6) is a measure developed to screen and identify individuals with low cancer health literacy (Dumenci et al., 2014). The CHLT-6 asks participants to answer six questions on topics related to cancer. Individuals are categorized as having inadequate or adequate cancer health literacy. Scores of five and above indicate adequate cancer health literacy and scores of four and below indicate inadequate health literacy (Kanu et al., 2021). The CHLT-6 has been found to be highly precise in identifying those with limited and adequate cancer health literacy (Dumenci et al., 2014).

The eHealth Literacy Scale (eHeals) (Norman and Skinner, 2006) is an 8-item measure developed to measure individuals combined knowledge, comfort and perceived skills at finding, evaluating and applying health information. Higher scores indicate higher eHealth literacy. In this study, the Cronbach’s alpha was 0.92 which is comparable to a study carried out with cancer caregivers and patients with prostate cancer with a Cronbach’s alpha of 0.94 (Song et al., 2017).

The General Self-Efficacy Scale (Schwarzer and Jerusalem, 1995) is a 10-item measure developed to assess individuals’ level of perceived self-efficacy. A higher score indicates higher self-efficacy. In this study, the Cronbach’s alpha was 0.90 indicating good internal consistency.

The Distress Thermometer (Roth et al., 1998) which is a single item instrument developed as a rapid screening tool for distress. Participants were asked to rate their distress level from 0 to 10 with higher scores indicating higher distress. The distress thermometer has been used among cancer caregivers previously (Kirk et al., 2022).

The Zarit Burden Interview Short Version (ZBI-12) measures caregiver burden and consists of 12 items (Bédard et al., 2001). Higher scores indicate higher caregiver burden. In this study, the Cronbach’s alpha was 0.85 indicating good internal consistency similar to a study carried out with patients and informal cancer caregivers which reported a Cronbach’s alpha of 0.93 (Barben et al., 2023).

### 2.4 Procedure

Surveys were available in online and hard-copy format, however no participants availed of the hard-copy option. Participants were presented with a plain language statement and consent form to read upon clicking the online link to access the survey. Participants were then presented with the socio-demographic questions described above and the HLCS-C, CHLT-6, eHeals, Self-Efficacy Scale, Distress Thermometer and the ZBI-12 in that order. Following the last question, participants were presented with a debriefing form which included contact details for a range of services that participants could avail of.

### 2.5 Data analysis

Statistical analyses were performed in IBM SPSS version 28. Frequency and descriptive statistics were conducted to describe the characteristics of the sample of cancer caregivers and their health literacy and eHealth literacy scores. Scores were calculated for each domain of the HLCS-C, the data indicated that HLCS-C scores were not normally distributed in our population. As a result, non-parametric tests were used to carry out the analysis. Mann–Whitney U tests and Kruskal-Wallis tests to examine the differences between groups based on socio-demographic characteristics across the HLCS-C domains and eHealth literacy scores. Chi-square tests were also used to investigate associations between socio-demographic characteristics and inadequate/adequate health literacy. Effect sizes were calculated to describe the magnitude of the difference in HLCS-C and eHeals scores between the groups, with scores between 0.2 and 0.5 considered small, 0.5 and 0.8 considered medium and >0.8 considered large according to Cohen (2013). Multiple linear regression analyses were performed to examine the association between each of the 10 domains of the HLCS-C, the CHLT-6, eHealth literacy and self-efficacy as the outcome. A logistic regression analysis was performed to examine the association between the 10 domains of the HLCS-C, the CHLT-6, eHealth literacy and caregiver burden. High caregiver burden and low caregiver burden were the binary outcomes, using the cut-off of 17 on the ZBI-12 to indicate high caregiver burden (Bédard et al., 2001). We performed a logistic regression to examine the association between the 10 domains of the HLCS-C, the CHLT-6, eHealth literacy and distress. High distress and low distress were the binary outcomes, using the cut-off point of 4 for the distress thermometer (Cutillo et al., 2017).

In addition to the above, mediation analyses were performed to test the mediating role of self-efficacy between health literacy/eHealth literacy and caregiver burden and distress. The bootstrapping method was used as recommended by Hayes (MacKinnon et al., 2004; Hayes, 2009) via Amos version 28. This method considers a mediator to have a mediational effect when the indirect effect of health literacy/eHealth literacy on burden/distress via self-efficacy is significant. The indirect effect is considered statistically significant if the bias corrected 95% confidence intervals around the indirect effect exclude zero. For this study we planned to examine six separate path models, (1) Indirect effect of cancer caregiver health literacy (HLCS-C) on burden via self-efficacy, (2) Indirect effect of cancer caregiver health literacy (HLCS-C) on distress via self-efficacy, (3) Indirect effect of inadequate/adequate health literacy on burden via self-efficacy, (4) Indirect effect of inadequate/adequate health literacy on distress via self-efficacy, (5) Indirect effect of eHealth literacy on burden via self-efficacy and (6) Indirect effect of eHealth literacy on distress via self-efficacy. Confirmatory factor analysis was applied using AMOS to test the fit indices of any latent variables in our path models. For the purposes of this study, there was only one latent variable based on the domain-based measure HLCS-C (Yuen et al., 2018). There are various fit indices available to assess the model fit, for this study we report the chi-square/df, the Comparative fit index (CFI), Incremental fit index (IFI), Tucker Lewis index (TLI), normed fit index (NFI) and Root Mean Square Error of Approximation (RMSEA). A cut-off point greater than 0.90 in the following indices CFI, IFI, NFI and TLI is considered an acceptable fit (Bentler and Bonett, 1980). A relative chi-square test (chi-square/df) with a value below 3 is considered an acceptable fit (Kline, 2011), while a value less than 0.08 is considered an acceptable fit for the RMSEA (MacCallum et al., 1996). The results of the confirmatory factor analysis indicated that the fit indices (Relative chi-square test = 3.871, CFI = 0.859, IFI =0.86, NFI - 0.821, TLI = 0.819, RMSEA = 0.118) did not confirm a satisfactory model fit. As a result, we did not examine the mediating effect of self-efficacy between caregiver health literacy (based on the HLCS-C domains) and burden or distress.

## 3 Results

### 3.1 Cancer caregiver characteristics

The final sample consisted of 208 informal caregivers. Sociodemographic characteristics for participants are shown in Table 1. The mean age of caregivers was 49 years (SD = 10.8) and ranged from 20 to 77 years. The characteristics of care recipients (i.e., cancer patients/survivors) as reported by cancer caregivers are also shown in Table 1.

**Table 1:** Cancer caregiver and care recipient characteristics.

**Caregiver characteristics**	**Frequency**	**%**
**Gender**		
Female	192	92.3
Male	15	7.2
Other	1	0.5
**Education level**		
Primary school	5	2.4
Secondary school	67	32.2
Third level	136	65.4
**Living area**		
Urban	115	55.3
Rural	90	43.3
Unsure	3	1.4
**Employment status**		
Employed	127	61.1
Unemployed	81	38.9
**Full-time caregiver**		
Yes	73	35.1
No	135	64.9
**Caregiver chronic illness**		
Yes	40	19.2
No	168	80.8
**Relationship to CR**		
Partner/Spouse	73	35.1
Child	60	28.8
Sibling	14	6.7
Parent	42	20.2
Extended family/close friends	19	9.1
**Time caring for CR**		
Less than one year	82	39.4
One to five years	100	48.1
More than five years	26	12.5

**Care recipient characteristics**	**Frequency**	**%**
**Care recipient cancer stage**		
Stage 1	7	3.4
Stage 2	13	6.3
Stage 3	28	13.5
Stage 4	108	51.9
Terminal	3	1.4
Different method of staging	9	4.3
Not staged	4	1.9
Unsure	36	17.3
**Care recipient treatment received**		
Chemotherapy	151	72.6
Radiotherapy	105	50.5
Surgery	118	56.7
Targeted therapy	47	22.6
Immunotherapy	34	16.3
Stem cell transplant	11	5.3
Alternative medicines	31	14.9
**Type of cancer**		
Female genital organs	10	4.8
Digestive organs	39	18.8
Male genital organs	9	4.3
Urinary tract	11	5.3
Lung	29	13.9
Brain	12	5.8
Breast	23	11.1
Lymphoma	9	4.3
Skin	5	2.4
Head, neck and thyroid	8	3.8
Leukemia	13	6.3
Sarcoma	3	1.4
Multiple Myeloma	2	1.0
Multiple cancers	26	12.5
Rare cancers	7	3.4

### 3.2 Cancer caregiver health literacy and eHealth literacy

Using the CHLT-6, 7% of cancer caregivers were in the inadequate health literacy category. Caregivers’ eHealth literacy scores ranged from 20–77 with a mean score of 28.56 (5.88). The mean scores for each HLCS-C domain are shown in Table 2. For the HLCS-C domains 1–8 (range 1–4), the highest mean score was 2.8 on the self-care domain. The lowest mean scores were for proactivity and determination to seek information, understanding caregiver needs and preferences, and social support. For the last two domains (range 1–5), scores were relatively high on both capacity to process information and active engagement with healthcare professionals.

**Table 2:** HLCS-C Scores.

**HLCS-C Domain**	**Mean (SD)**	**Range**
Proactivity and determination to seek information	1.8 (0.7)	4–16
Adequate information about cancer and cancer management	2.2 (0.6)	4–16
Supported by healthcare professionals to understand information	2.1 (0.7)	5–20
Social support	1.9 (0.7)	4–16
Cancer-related communication with care recipients	2.1 (0.8)	4–16
Understanding care recipient needs and preferences	1.8 (0.5)	6–19
Self-care	2.8 (0.6)	5–20
Understanding the healthcare system	2.1 (0.6)	6–24
Capacity to process information	3.7 (0.8)	4–20
Active engagement with healthcare professionals	3.6 (0.9)	4–20

### 3.3 Caregiver characteristics, health literacy and eHealth literacy

Health literacy scores significantly differed by gender across three HLCS-C domains. Scores were higher for males (*Md =* 2, *n =* 15) than for females (*Md =* 1.75, *n =* 192) on ‘Proactivity and determination to seek information’ *U* = 2035, *z* = 2.72, *p* < 0.05, *r* = 0.2. In contrast, females scored higher than males on ‘Cancer-related communication with the care recipient’, females (*Md =* 2, *n =* 192) and males (*Md =* 1.25, *n =* 15), *U* = 965.5, *z* = −2.14, *p* < 0.05, *r* = 0.2 and ‘Self-care’, [females (*Md =* 2.8, *n =* 192) and males (*Md =* 2.4, *n =* 15), *U* = 690, *z* = −3.38, *p* < 0.01, *r* = 0.2].

Cancer caregivers who were currently working (*Md =* 1.83, *n =* 127) scored significantly higher on one domain ‘Understanding care recipient needs and preferences’ compared to those who were not working (*Md =* 1.67, *n =* 81), *U* = 4307, *z* = −2.0, *p* < 0.05, *r* = 0.1.

The scores on the domain ‘Capacity to process information’ differed according to the caregivers’ relationship to the care recipient, *x^2^* (4, *n* = 208) = 11.80, *p* < 0.05. Post-hoc tests in which a Bonferroni adjustment was applied indicated that there was a significant difference between extended family/close friends (*Md* = 4) and children (*Md* = 3.5) and partner/spouse (*Md* = 3.75). Scores on the domain ‘Active engagement with healthcare providers’ also differed according to the caregivers’ relationship to the care recipient *x^2^* (4, *n* = 208) = 9.172, *p* < 0.05. However, following post-hoc comparisons in which a Bonferroni adjustment was applied, no significant differences remained between any of the groups. There were no significant differences (*p* > 0.05) regarding cancer caregiver education level, living area, health status, time spent caring, caregiver status, cancer stage or number of treatments received by the care recipient or cancer type across health literacy domains.

Regarding the CHLT-6, a significant association between education level and Adequate/Inadequate health literacy was observed *x*^2^(2) = 13.94, *p* < 0.001, Cramer’s *V* = 0.30 indicating a medium effect size, with higher education level being associated with adequate health literacy. Additionally, an association between living area and Adequate/Inadequate health literacy was observed *x*^2^(1) = 4.623, *p* < 0.05, Cramer’s *V* = 0.15, with living in an urban area being associated with adequate literacy. No other significant associations (*p* > 0.05) were observed between any other socio-demographic variables and the CHLT-6 scores.

eHealth literacy scores significantly differed with cancer caregivers’ education level, *x^2^* (2, *n* = 205) = 8.31, *p* < 0.05. Post-hoc analysis using the Bonferroni correction for multiple tests indicated that caregivers who had up to a primary school education scored significantly lower than caregivers who had third level or above (*p* < 0.05), *r* = 0.6. There was a significant difference in eHealth literacy scores between caregivers currently working (*Md* = 31, *n* = 125) and those who were not (*Md* = 27, *n* = 80), *U* = 3946.5, *z* = −2.551, *p* < 0.05, *r* = 0.02. Additionally, eHealth literacy scores significantly differed between caregivers who were fulltime caregivers (*Md* = 26, *n* = 72) and part-time caregivers (*Md* = 31, *n* = 133), *U* = 6287.0, *z* = 3.709, *p* < 0.001, *r* = 0.3. Caregivers eHeals scores were correlated with caregiver age (*r* = −0.18, *n* = 205, *p* < 0.001) indicating a weak negative association between older age and lower eHeals scores. No other socio-demographic variables were associated with the eHealth literacy scores.

### 3.4 Health literacy, eHealth literacy and self-efficacy

Caregivers reported a mean self-efficacy score of 30.70 (5.02, 12–40). For our self-efficacy model (Table 3), the health literacy and eHealth literacy variables accounted for 25% of variance of caregiver self-efficacy scores, *F*(12, 192) = 5.22, *p* < 0.001. The following health literacy domains independently predicted self-efficacy among caregivers: *cancer-related communication with the care recipient* and *Understanding care recipient needs.* Understanding care recipient needs and preferences had the strongest impact on self-efficacy (*B* = −1.70; CI, −3.22 to −0.18; *p* < 0.05). eHealth literacy also independently predicted caregiver self-efficacy (*B* = 0.25, CI, 0.11 to 0.38; *p* < 0.01).

**Table 3:** Cancer caregiver health literacy, eHealth literacy and self-efficacy.

	**Self-efficacy (GSE)**					
				**95% CI**		
**HL domain**	***B***	**SE**	**β**	**Lower**	**Upper**	***R*** ^**2**^
Proactivity and determination to seek information	−0.66	0.50	−0.90	−1.64	0.33	0.25***
Adequate information about cancer and cancer management	0.43	0.80	0.05	−1.13	1.98	
Supported by HCPs to understand information	0.82	0.67	0.12	−0.50	2.15	
Social support	−0.55	0.56	−0.07	−1.66	0.55	
Cancer-related communication with the care recipient	−1.05*	0.53	−0.16	−2.09	−0.01	
Understanding care recipient needs and preferences	−1.70*	0.77	−0.18	−3.22	−0.18	
Self-care	0.06	0.59	0.01	−1.10	1.21	
Understanding the healthcare system	0.03	0.73	0.00	−1.40	1.45	
Capacity to process health information	0.29	0.62	0.04	−0.93	1.52	
Active engagement with HCPs	0.40	0.54	0.07	−0.66	1.47	
CHLT-6	−0.57	1.31	−0.03	−3.15	2.02	
eHealth Literacy	0.25***	0.07	0.29	0.11	0.38	

*p < 0.05, **p < 0.01, ***p < 0.001.

### 3.5 Health literacy, eHealth literacy, caregiver burden and distress

Cancer caregivers reported a caregiver burden score of 20.83 (8.42, 0–44). In this sample, 67% of cancer caregivers had high levels of burden. A logistic regression (Table 4) was performed to ascertain the effects of 10 health literacy domains, having inadequate or adequate health literacy and eHealth literacy on the likelihood of caregivers having high caregiver burden. The logistic regression model was statistically significant *x*^2^(12) = 49.11, *p* < 0.001. The model as a whole explained between 21.3 and 29.6% in high caregiver burden, correctly classifying approximately 74.1% of cases. Increasing eHealth literacy and increasing caregivers’ capacity to understand the care recipients needs and preferences were associated with an increased likelihood of exhibiting high caregiver burden.

**Table 4:** Cancer caregiver health literacy, eHealth literacy, burden and distress.

	Distress (NCCN distress thermometer)							Caregiver burden (ZBI-12)						
**Variable**	***B***	**SE**	**Wald**	**df**	**Odds Ratio**	**95% CI for OR**		***B***	**SE**	**Wald**	**df**	**Odds Ratio**	**95% CI for OR**	
**Proactivity and determination to seek information**	−0.75*	0.29	6.69	1	0.47	Lower	Upper						Lower	Upper
						0.27	0.83	−0.02	2.6	0.01	1	0.98	0.59	1.64
**Adequate information about cancer and cancer management**	0.41	0.47	0.75	1	1.50	0.60	3.77	0.69	0.45	2.40	1	2.0	0.83	4.81
**Supported by HCPs to understand information**	0.14	0.40	0.12	1	1.15	0.52	2.53	0.04	0.37	0.01	1	1.04	0.51	2.14
**Social support**	0.60	0.35	2.73	1	1.79	0.90	3.57	0.13	0.29	0.20	1	1.14	0.64	2.02
**Cancer-related communication with the care recipient**	−0.45	0.31	2.06	1	0.64	0.35	1.18	0.40	0.30	1.76	1	1.48	0.83	2.65
**Understanding care recipient needs and preferences**	1.10*	0.48	5.27	1	3.0	1.17	7.61	1.37**	0.45	9.46	1	3.94	1.64	9.43
**Self-care**	−0.02	0.35	0.00	1	0.99	0.50	1.96	0.59	0.32	3.40	1	1.81	0.96	3.40
**Understanding the healthcare system**	1.04*	0.47	4.97	1	2.83	1.13	7.05	−0.76	0.40	3.62	1	0.47	0.21	1.02
**Capacity to process health information**	−0.11	0.37	0.09	1	0.90	0.44	1.85	−0.14	0.34	0.17	1	0.87	0.45	1.68
**Active engagement with HCPs**	0.53	0.33	2.67	1	1.71	0.90	3.24	−0.20	0.30	0.45	1	0.82	0.46	1.46
**eHealth literacy**	0.07	0.04	3.39	1	10.7	1.00	1.16	0.08*	0.04	4.06	1	1.08	1.0	1.17
**CHLT-6**	0.10	0.74	0.02	1	1.10	0.26	4.70	0.88	0.63	1.95	1	2.42	0.70	8.36

*p < 0.05, **p < 0.01, ***p < 0.001.

Caregivers reported a mean distress score of 5.85 (2.80, 0–10). The prevalence of high distress among cancer caregivers was 78%. Our second model (Table 4), investigating the effects of the 10 health literacy domains, having adequate or inadequate health literacy and eHealth literacy on the likelihood that caregivers have high distress, was statistically significant *x*^2^(12) = 37.22, *p* < 0.001. The model explained between 16.6 and 25.7% of variance in high distress levels, correctly classifying approximately 84.4% of cases. Decreasing caregiver capacity in proactivity and determination to seek information was associated with an increased likelihood in exhibiting high distress. Increasing caregiver capacity in understanding the care recipient preferences and needs and understanding the healthcare system was associated with an increased likelihood in exhibiting high distress.

### 3.6 The mediating role of self-efficacy

**Figure 1:**
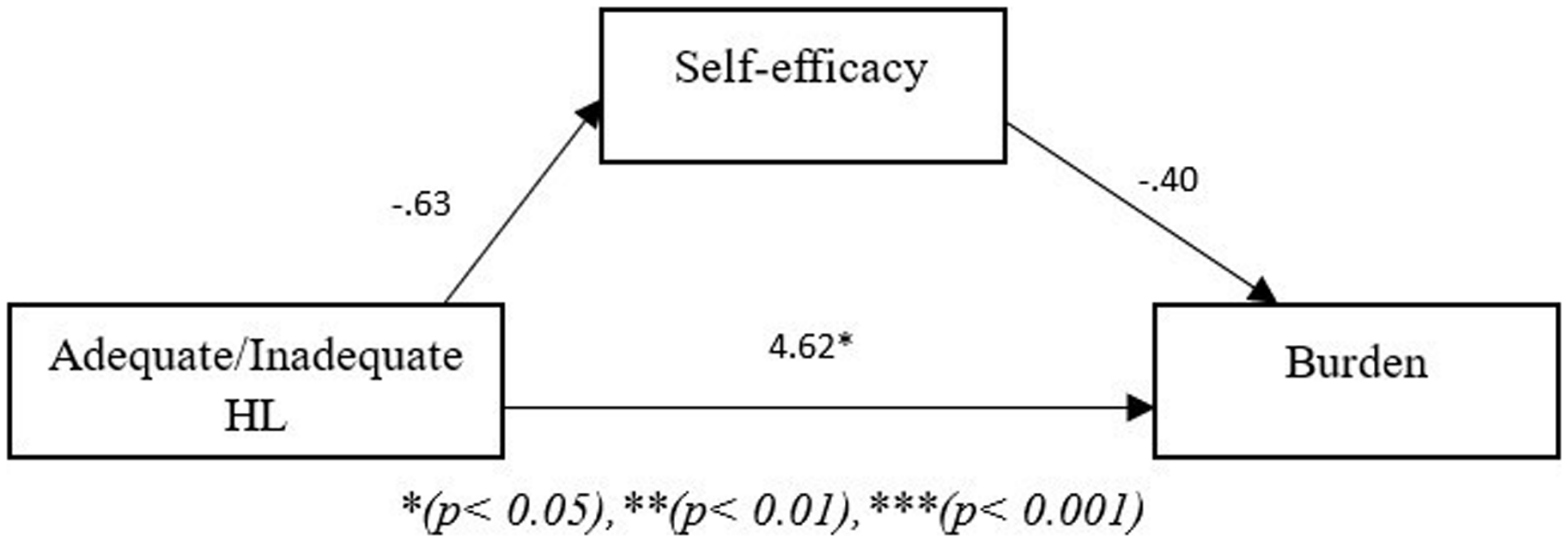
Path model showing mediating role of self-efficacy between health literacy and burden. *p < 0.05, **p < 0.01, ***p < 0.001.

**Figure 2:**
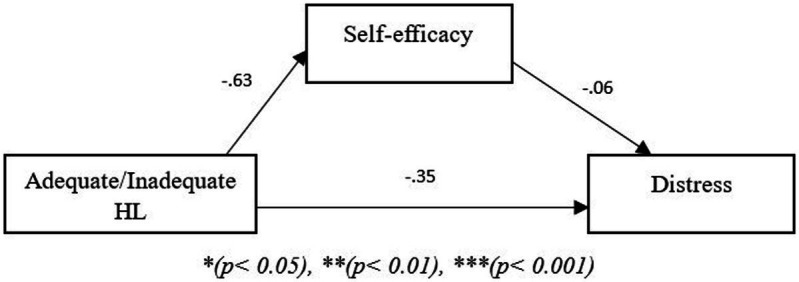
Path model showing mediating role of self-efficacy between health literacy and distress. *p < 0.05, **p < 0.01, ***p < 0.001.

The models in the figures below show two path models where the independent variable is Adequate/Inadequate health literacy, the mediating variable is self-efficacy and the dependent variable is either burden (Figure 1) or distress (Figure 2). In the first model, the results revealed that self-efficacy did not mediate the relationship between Adequate/Inadequate health literacy and burden (*B* = −0.25, *p* > 0.05) but that there was a direct effect of Adequate/Inadequate health literacy on burden (*B* = 4.62, *p* < 0.05). The second path model showed that self-efficacy did not mediate the relationship between Adequate/Inadequate health literacy and distress (*B* = 0.04, *p* > 0.05) nor was a direct relationship evident between Adequate/Inadequate health literacy and distress (*B* = −0.351, *p* > 0.05).

**Figure 3:**
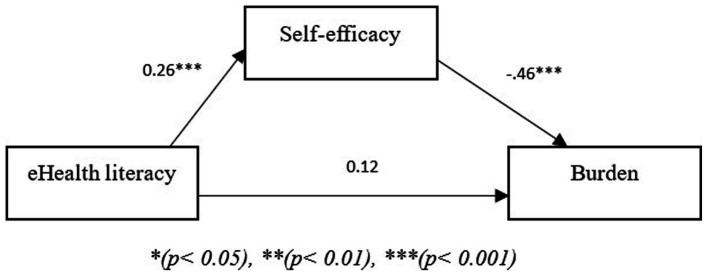
Path model showing mediating role of self-efficacy between eHealth literacy and burden. *p < 0.05, **p < 0.01, ***p < 0.001.

**Figure 4:**
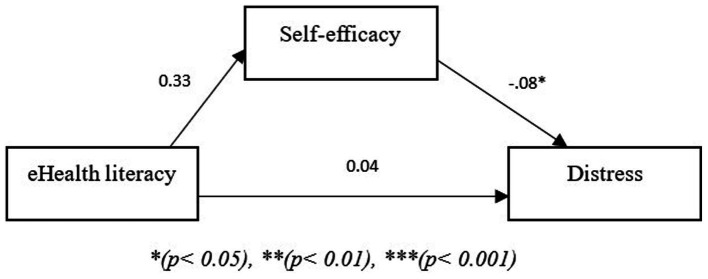
Path model showing mediating role of self-efficacy between eHealth literacy and distress. *p < 0.05, **p < 0.01, ***p < 0.001.

The models shown in the figures below show two path models where the independent variable is eHealth literacy, the mediating variable is self-efficacy and the dependent variable is burden (Figure 3) or distress (Figure 4). In the third model, the results showed that self-efficacy mediated the relationship between eHealth literacy and burden (*B* = −0.15, *p* < 0.05). Additionally, given that there was no direct effect of eHealth literacy on burden (*B* = 0.12, *p* < 0.05) the relationship between eHealth literacy and burden is fully mediated through self-efficacy. The fourth path model with distress as the dependent variable showed that self-efficacy also mediated the relationship between eHealth literacy and distress (*B* = −0.0.02, *p* < 0.05). Additionally, there was no direct effect of eHealth literacy on distress (*B* = 0.043, *p* > 0.05); thus, self-efficacy fully mediated the relationship between eHealth literacy and distress.

## 4 Discussion

Despite the importance of cancer caregiver health literacy, little research has been undertaken to investigate the levels of health literacy among cancer caregivers and the factors associated with cancer caregiver health literacy. To the best of the authors knowledge this is the first study to explore cancer caregiver health literacy and eHealth literacy within the same study. Furthermore, we provided a more nuanced and comprehensive understanding of cancer caregiver health literacy by measuring adequate/inadequate health literacy and health literacy across multidimensional domains. The findings of the present study suggest low levels of inadequate health literacy but variability across 10 health literacy domains in this sample of cancer caregivers. Furthermore, our findings indicate that there were differences in caregivers’ scores on some of these domains according to caregiver gender, employment status, relationship to the care recipient, education level and living area across (‘*Proactivity and determination to seek information,*’ ‘*Cancer-related communication with the care recipient,*’ ‘*Self-care’* and ‘*Understanding care recipient needs and preferences’*) and CHLT-6 scores. Additionally, we found differences across eHealth literacy scores according to education level, employment status and caregiver status. We found different health literacy domains were associated with caregiver self-efficacy (‘*Cancer-related communication with the care recipient*’ and ‘*Understanding care recipients needs and preferences*’), burden (‘*Understanding care recipient needs and preferences*’) and distress (‘*Proactivity and determination to seek information’*, ‘*Understanding care recipient needs and preferences’* and ‘*Understanding the healthcare system*’), while eHealth literacy was associated with self-efficacy and burden.

There is little research against which to compare the level of adequate/inadequate health literacy in this sample. Although similar to a study of informal caregivers (Oakley-Girvan et al., 2023), a previous study carried out with cancer caregivers found a much higher proportion of cancer caregivers had limited health literacy compared to the current sample (Gabriel et al., 2021). This contrast may be explained by that study being conducted in a developing country with limited cancer services which may explain the poor health literacy among cancer caregivers (Gabriel et al., 2021).

The use of a second health literacy measure, the HLCS-C which is more multidimensional in nature enabled the identification of key health literacy strengths and difficulties among our sample of cancer caregivers adding to a more nuanced understanding of health literacy among cancer caregivers. Cancer caregivers scored highest on the *Self-care*, *Capacity to process health information* and *Active engagement with healthcare professionals.* Our findings indicate that there were high self-care scores across the board however, a gender difference indicates that self-care is higher in females than males. This may suggest that self-care is an element which specifically needs to be targeted in men. Additionally, previous research has indicated that in the context of cancer caregiving male caregivers may be at higher risk of carrying out unhealthy behaviors such as smoking, binge drinking or physical activity (Cho et al., 2020). Cancer caregivers who were extended family or close friends to the care recipient scored higher than those who were children or a partner in their capacity to process information. This may be down to the closer relationship that children and partners may have to the care recipient, for example family members often experience an increase in distress throughout the cancer trajectory particularly just after diagnosis (Lavallée et al., 2018) which impact the caregivers’ ability to process information.

In contrast, cancer caregivers scored lowest on the *Proactivity and determination to seek information, social support* and *Understanding care recipient needs and preferences*. Low scores in caregivers proactivity to seek information may prove problematic for the caregiver role as cancer patients rely on the caregiver to gather relevant information from different sources to help with making health related decisions (Given et al., 2012). Overall caregivers scored low on *Understanding care recipient needs and preferences*, however caregivers who were employed scored higher in this domain than those who were not working which may highlight a particular need to target unemployed caregivers with interventions which aim to improve their ability to understand the needs of the care recipient. Low scores in the *Social support* domain suggest that caregivers feel isolated and have difficulty identifying at least one individual who can provide support. Previous research has shown that caregivers preferred to go to their spouses for support; however, when a spouse was not available caregivers preferred to be independent in regard to their support needs (Nightingale et al., 2016). A large proportion of the participants in our sample were providing care for their spouse/partner, consequently their partners may not have been able to provide the support the caregiver needed which may reflect some of the low scoring in the social support domain.

In regard to caregiver eHealth literacy, younger age and higher education level was associated with higher eHealth literacy scores. Previous research has shown that older adults have lower eHealth literacy (Jensen et al., 2010) which may be of particular concern as the online world is fast becoming a primary medium for accessing health information.

We found that 78% of cancer caregivers in this study were experiencing high distress. We found that increased capacity to seek information was associated with decreased distress in our sample. Research has previously indicated that information helps caregivers to cope by reducing feelings of uncertainty (Loke et al., 2003) which could explain the decrease in distress when caregivers ability to find relevant information increases. In contrast, increased capacity in understanding of care recipient needs and preferences and understanding the healthcare system was associated with increased distress. Caregiver distress is a result of caregiver capacity to cope with the demands of caregiving (Applebaum et al., 2022) thus, it is possible that increasing caregivers understanding in both of these areas may increase their comprehension of the demands of their role which may increase their distress.

In our study we found 67% of cancer caregivers reported high levels of caregiver burden. Increased eHealth literacy was associated with an increased likelihood of experiencing high burden in our sample of cancer caregivers. Access to eHealth sources and more information may increase the prevalence of information overload in caregivers which could explain an increase in burden. Research indicates that information overload may impact caregiver decision making ability and result in ineffective practices which impact caregiving outcomes (Kim, 2021). Additionally, previous research has indicated that searching online for cancer related information confronts patients and caregivers with negative disease information which may have a negative impact on their wellbeing (Ginossar, 2008) which may in turn increase their burden. Increased scores in the *Understanding care recipient needs and preferences* domain was also associated with an increased likelihood of exhibiting high distress. One explanation for this could be due to the high number of late-stage cancer patients in our sample.

The average self-efficacy levels of caregivers in this sample are similar to those reported among caregivers in previous studies (Johansen et al., 2018; van Hof et al., 2023). Increased eHealth literacy was associated with increased self-efficacy and an increased likelihood of experiencing high burden among our sample of cancer caregivers. Caregivers often turn to eHealth sources for necessary information which they do not already have (Kim et al., 2019) which may result in caregivers feeling that they are better able to carry out their tasks for the care recipient thus leading to increased health literacy. An increase in caregivers ‘*cancer-related communication with the care recipient’* was associated with decreased self-efficacy. High capacity in cancer-related communication with the care recipient suggests that the communication is of high quality consisting of honest and open discussions about the cancer, outcomes, information received at from healthcare professionals and the care recipients’ health (Yuen et al., 2018). Open conversations consisting of these elements may result in the caregiver having a better understanding of the care recipient’s prognosis and needs and thus may have an increased understanding of what may be expected of them, thus resulting in lower self-efficacy. Additionally, an increase in ‘*Understanding care recipient needs and preferences’* was associated with a decrease in self-efficacy. One explanation for this could be the evidence which indicates that informal caregivers often lack the necessary training to carry out the caregiving tasks (van Ryn et al., 2011). Further, the more caregivers understand the needs of the care recipient the more caregivers realize the training they require and thus result in decreased self-efficacy due to feeling unable to meet the care recipient’s needs. Additionally, we found that self-efficacy played a mediating role between eHealth literacy and caregiver outcomes but not between adequate/inadequate health literacy and caregiver outcomes. Understanding the role of self-efficacy in this context, may be particularly important as previous research has shown that heightened caregiver self-efficacy is associated with lower levels of burden (Phongtankuel et al., 2023) and lower levels of distress (Chirico et al., 2017), while earlier research indicates that health literacy may act as a barrier to self-efficacy (Berkman et al., 2011). Further, to have the confidence to perform a behavior, an individual must first understand what the necessary behavior is (Berkman et al., 2011). Thus, cancer caregivers with higher eHealth literacy may have more confidence in their ability to carry out the caregiving role which may in turn impact caregivers’ burden and distress levels.

### 4.1 Strengths and limitations

A strength of our study is the use of two health literacy measures, the CHLT-6, a more objective measure of health literacy levels and the HLCS-C which is a comprehensive self-report health literacy measure that allows the identification of specific health literacy needs. If we had only used the CHLT-6 which focuses primarily on the skill of the individual we would have concluded that caregivers in this population had mostly adequate levels of health literacy; however, scores from the HLCS-C indicate that there are key areas of need regarding cancer caregiver health literacy and these areas differed by demography. Applying the population specific measurement tool among cancer caregivers, may have also aided in capturing elements of cancer caregiver health literacy specifically which may have previously been missed (Moore et al., 2021).

Regarding limitations, the cross-sectional nature of this study precludes any conclusions regarding the causality of any of the associations that were observed. Longitudinal studies which measure cancer caregiver health literacy across multiple timepoints would provide better insight into cancer caregivers’ health literacy needs; as health literacy is context specific (Sørensen et al., 2012), cancer caregivers needs may change as their roles and responsibilities change across the cancer trajectory. It should also be noted that participants self-selected to take part in the survey and all participants accessed and completed the survey online. It is worth noting the level of burden and distress reported by caregivers in this sample was high; thus, this may have played a role in why caregivers decided to participate. Furthermore, this sample cannot be said to be representative of all cancer caregivers as the majority of caregivers were female, employed, had a high level of education, cared for an individual with stage 4 cancer and most were providing care to a partner or a child. Further work is needed to capture health literacy across a more diverse sample of caregivers. It is also worth noting the poor fit of the 10 domains of the HLCS-C as the latent construct of cancer caregiver health literacy; this may indicate and support recent arguments for examining health literacy in a domain-based format rather than as a univariate construct as health literacy encompasses many factors. As such, future research investigating the mediating effect of self-efficacy on the relationship between the domains of health literacy and relevant health outcomes would be beneficial. Last, the path models used in the current sets of mediation analyses were derived from cross-sectional data, thus longitudinal studies are required to confirm the causal pathways.

### 4.2 Clinical implications

Our findings have provided insight into the complexity of health literacy among cancer caregivers, including their difficulties with obtaining information, understanding care recipient preferences, and engaging with their social supports. Identification of the strengths and weaknesses regarding cancer caregiver health literacy will help healthcare professionals to provide caregivers with increased support while providing care across the cancer trajectory. The identification of these areas of difficulty can inform the future development of tailored interventions to support caregivers to build those health literacy capacities. Research is currently being conducted in areas related to improving similar health literacy capacities among cancer caregivers as those which caregivers found challenging in our sample; for example, the development of a communication and health literacy curriculum for caregivers (Wittenberg et al., 2020) and the development of a psychological support intervention to increase cancer caregivers and patients ability to make health decisions, deal with conflicts and alleviate distress (Cincidda et al., 2022). Given the potentially negative impact that low caregiver health literacy may have on cancer survivor outcomes, it would seem imperative to develop effective health literacy interventions which enable caregivers to build their skills and enable this group to better support those with cancer as a result.

Understanding the relationship between cancer caregiver health literacy and socio-demographics provides key insights for targeting potentially at-risk groups, for example research could potentially address the different health literacy needs of males and females to ensure that interventions can be appropriately tailored. Additionally, understanding the relationship between cancer caregiver health literacy and varying caregiver outcomes, such as burden and distress may be particularly important given the impact that caregiver outcomes can have on patient outcomes.

Our findings indicate particularly high levels of burden and distress in this sample of cancer caregivers. Such high levels of caregiver burden and distress may impede the quality-of-care caregivers are able to provide, potentially impacting health outcomes for both survivors and caregivers. It may be worth noting, as this survey was conducted post COVID-19 there may be a particular need to understand the impact that this major life event had on cancer caregiving particularly given these high levels of distress and burden. Additionally, the introduction of routine screening for burden and distress would make healthcare professionals aware of burden and distress, allowing healthcare professionals to respond appropriately. Cancer caregivers who took part in a previous study in which they completed a distress screener, reported that using the caregiver-focused distress screening tool was a positive experience and reported feeling validated in the importance of their role (Shaffer et al., 2019). Routine screening would allow healthcare professionals to identify caregivers who may be experiencing high levels of distress or burden because of the caregiving role and to further help support caregivers in need, which in turn has the potential to improve quality of care. Furthermore, given the relationship between aspects of cancer caregiver health literacy and caregiver burden and distress, looking at ways to alleviate high levels of burden and distress by addressing caregiver health literacy skills may be an area that warrants attention during clinical consultations when caregivers are present and when their role is critical to patient care and recovery.

Caregivers often use the online world to obtain health information and aid with making healthcare decisions (Vasquez et al., 2022). The utilization of digital health resources online has been found to improve the understanding of information and reduce unmet needs (Bamgboje-Ayodele et al., 2021). Thus, understanding cancer caregiver eHealth literacy is highly important. The findings in our study indicate that caregivers reported high eHealth literacy scores, however potentially eHealth literacy among older caregivers may be an area which could be addressed. Additionally, given that the majority of participants in our sample were internet users, it may be useful to look at caregivers who may not use the internet or have access to the internet who may have lower eHealth literacy levels, which could prove problematic particularly given the increased development of digital options for the delivery of information following COVID-19 (Dionne-Odom et al., 2022).

## 5 Conclusion

This is the first study to examine the relationship between cancer caregiver health literacy and caregiver socio-demographic characteristics. Additionally, this is also the first study to investigate the relationship between cancer caregiver health literacy and caregiver outcomes, indicating that there are aspects of health literacy which have more impact on caregiver outcomes than others. The high level of distress and burden among this population, and the potential role that health literacy might play warrants attention. The findings from this study will inform future development of tailored interventions to aid with improving health literacy among caregivers to better support them in their pivotal role. Through a greater understanding of health literacy and eHealth literacy among cancer caregivers, future research can target key areas of need such as, caregivers’ capacity to seek out relevant information, understanding the needs of the care recipient and the available social support for caregivers.

## Data availability statement

The original contributions presented in the study are included in the article/supplementary materials, further inquiries can be directed to the corresponding author.

## Ethics statement

The studies involving humans were approved by Dublin City University Research Ethics Committee. The studies were conducted in accordance with the local legislation and institutional requirements. The participants provided their written informed consent to participate in this study.

## Author contributions

CM: Writing – original draft, Writing – review & editing. PG: Supervision, Writing – review & editing. SD: Supervision, Writing – review & editing.
